# Ischemic Stroke as the Presenting Feature for Non‐acute Promyelocytic Leukemia Variant of Acute Myeloid Leukemia: A Case Report

**DOI:** 10.7759/cureus.41083

**Published:** 2023-06-28

**Authors:** Priya Pankaj, Chinmaya K Panda, Habib Md R Karim, Saroj Bala, Aditya Bidwaikar

**Affiliations:** 1 Anaesthesiology, Critical Care, and Pain Medicine, All India Institute of Medical Sciences, Raipur, Chhattisgarh, IND; 2 Clinical Haematology, All India Institute of Medical Sciences, Raipur, Chhattisgarh, IND

**Keywords:** embolectomy, acute management, clinical features, acute myeloid leukemia (aml), cerebro-vascular accident (stroke)

## Abstract

Acute myeloid leukemia (AML) patients encounter complications mainly due to their underlying disease or chemotherapy. Although they are at high risk for both hemorrhagic and thrombotic complications, thrombotic vascular complication as an initial manifestation is less common and rarely reported, especially in non-acute promyelocytic leukemia (non-APML). A 58-year-old female with no co-morbidity presented with fever, decreased appetite, headache, and weakness in her left upper and lower limbs. Laboratory findings showed hyperleukocytosis with 90% blast cells and thrombocytopenia (50,000/dl). While investigated and conservatively managed, she developed a seizure and loss of consciousness on the same day and was admitted to the intensive care unit. Computed tomography showed a massive right infarct in the middle cerebral artery territory with a significant midline shift. Flow cytometry indicated the diagnosis of non-APML; chemotherapy, platelet transfusion, unfractionated heparin, mechanical ventilation, and other supportive treatments were started. While managing this case, we faced challenges in decision-making on thrombolysis, craniotomy, and chemotherapy. The case highlights the salient points and dilemmas in managing such an acutely ill patient in critical care.

## Introduction

Thrombotic events are strongly associated with cancers, especially solid malignancies, and lymphoma [1]. These manifestations are also reported in acute myeloid leukemia (AML), but thrombotic incidents are scarce as the presenting complaints or manifestations, especially in the non-acute promyelocytic leukemia variant. Although the presentation is variable, patients with AML mostly present with fever, weakness, anemia, infections, and bleeding [2]. There is also an increased incidence of cardiovascular and cerebrovascular events like heart failure, ischemic heart disease, coronary artery disease, pulmonary embolism, and stroke in AML patients [3]. Acute leukemia patients can present with localized thrombosis or disseminated intravascular coagulation during the disease. Thrombotic manifestations as an initial presentation before the diagnosis of AML are rarely reported [4]. We present a case of massive ischemic stroke in a patient before the diagnosis of AML, a non-acute promyelocytic leukemia (non-APML) variant.

## Case presentation

A 58-year-old female (58 kg, 162 cm) with no known co-morbidities presented with a weak history of low-grade fever with chills and rigor, decreased appetite, body pain, and headache. She had stable hemodynamics with left hemiparesis at presentation. Other physical examinations were unremarkable except few palpable cervical lymph nodes. Complete blood count showed hyperleukocytosis (1,56,600/mm^3^), thrombocytopenia (47000/mm^3^), and hemoglobin of 7.1 g/dL. Her peripheral blood smear showed 85-90% blast cells. The liver, renal, coagulation, and serum electrolyte profile were within normal range. Malaria immune histochemistry and dengue NS1 and NS2 antigens were negative. Her urine routine and microscopy had shown an abundance of pus cells (35-40 cells/High power field).

**Figure 1:**
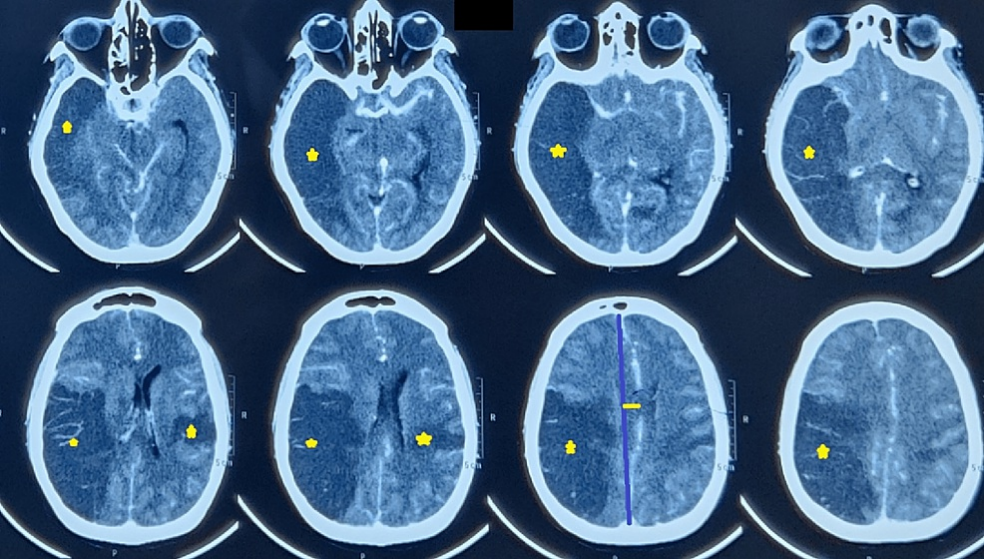
Non-contrast computed tomography images of the brain Non-contrast computed tomography brain demonstrates infarction (stars) in the right middle cerebral artery distribution, left parietal lobe, with mass effect and midline shift (depicted using straight lines).

In suspicion of hematopoietic malignancy, a flow cytometry panel was sent. On the next day, there was a sudden onset of mental obtundation, chest discomfort, and desaturation, followed by an episode of seizure was observed. The seizure was controlled with intravenous midazolam and levetiracetam. Arterial blood gas and electrocardiography were normal except for sinus tachycardia. The airway was secured, given the Glasgow Coma Scale (GCS) was E2V2M4, and was shifted to the intensive care unit (ICU) for further management. Assessment of the National Institute of Health Stroke Scale (NIHSS) scored 32. Non-contrast computerized tomography of the head showed a massive right middle cerebral artery territory infarct (Alberta stroke program early computed tomography score of 3) and left parietal lobe infarct with a mass effect of an 8.6 mm midline shift (Figure 1).

**Figure 2:**
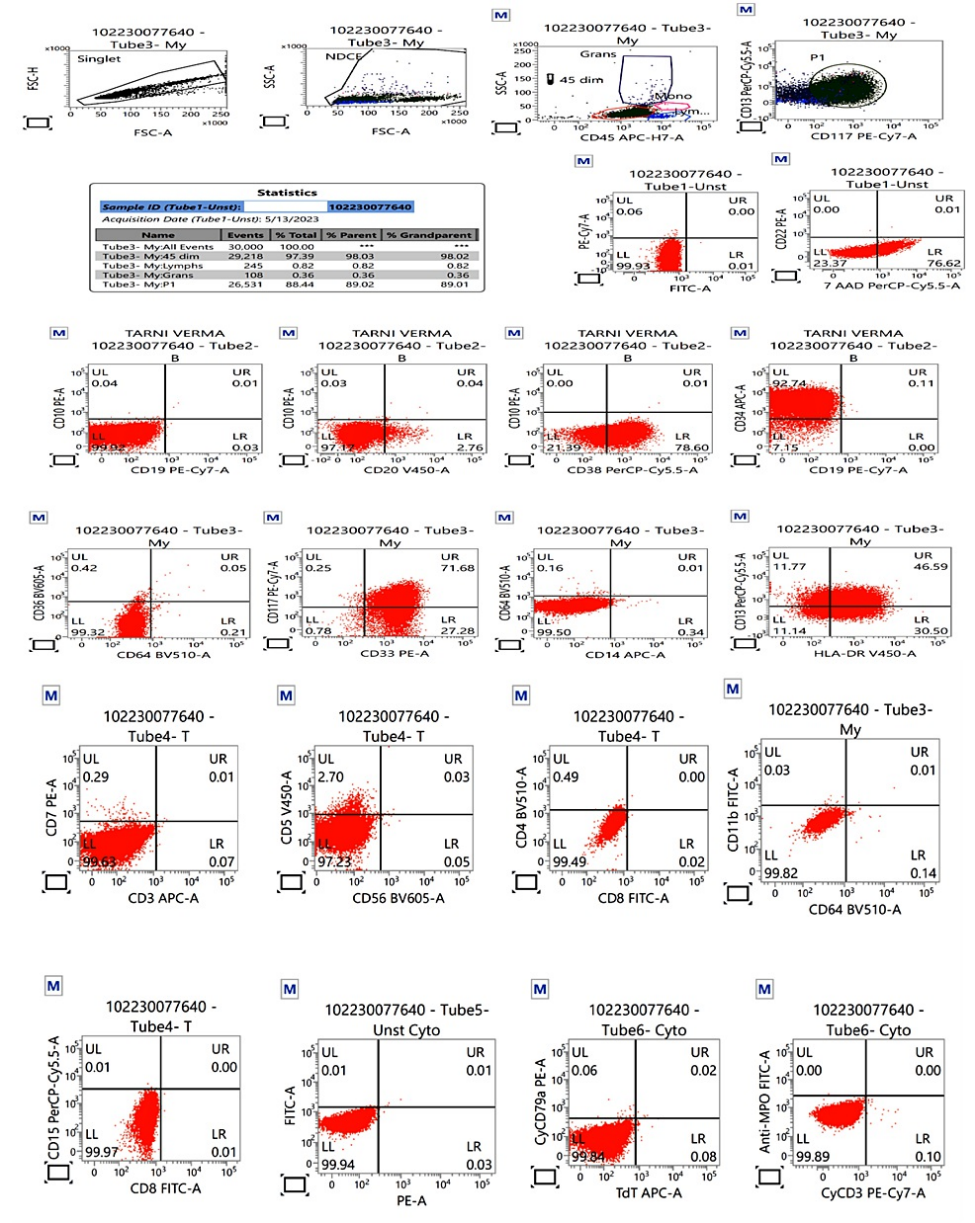
Flow cytometry results Flow cytometry showing acute myeloid leukemia (non-APML variant on immunophenotyping), positive for CD13, CD33, CD34, CD38, CD117, and HLA-DR. non-APML: non-Acute promyelocytic leukemia

Anti-edema measure was initiated with 3% normal saline. Considering the limb weakness days before the presentation, thrombocytopenia, and high NIHSS score, i.e., 26, intravenous tissue plasminogen activator administration was withheld. Unfractionated heparin (UFH) 5000 IU (international unit) intravenously thrice daily was started to prevent thrombosis, keeping activated partial prothrombin time (aPTT) twice that of the control as the target. Her GCS decreased to E1V(T)M2 with bilateral pupil dilatation and absent pupillary reflex. Decompressive craniotomy was deferred because of thrombocytopenia even after transfusion. The flow cytometry showed CD13, CD33, CD34, CD38, CD117, and HLA-DR positivity. Immunophenotypic analysis on CD45 versus side scatter had revealed 88.4% of blasts that exhibited a CD45 dim to moderate expression. The blasts depicted a moderate to bright CD34, moderate CD38, CD117, CD33, HLA-DR, and dim CD13. Blasts were negative for cytoplasmic myeloperoxidase (Figure 2).

**Figure 3:**
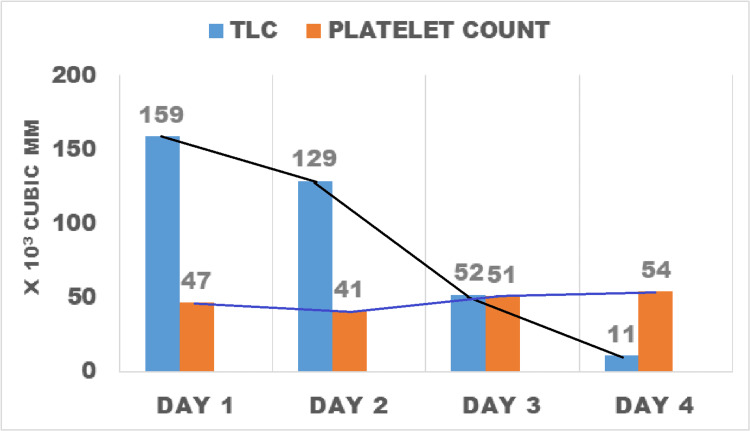
Bar diagram with connected lines showing leucocyte and platelet counts over the four days of hospitalization.

These findings narrowed the diagnosis to non-acute promyelocytic leukemia (non-APML) with monocytic predominance. Platelets were transfused daily but could maintain a count of 40,000-50,000/mm^3^ (Figure 3).

Induction chemotherapy with hydroxyurea and cytarabine was started. Adequate hydration and normothermia were maintained preemptively for tumor lysis syndrome. Despite the conservative approach, she showed no improvement in neurological status. On day-4 of ICU admission, she expired.

## Discussion

Patients with AML encounter many complications, either due to their underlying disease or related to the administration of chemotherapy. Studies indicate that AML patients have a 2-year cumulative venous thrombosis incidence of 5.4% [5]. Thrombotic vascular complication as an initial manifestation is less common, which makes this discussion worthwhile. In a case series of 24 acute leukemia patients, only 3.4% of non-M3 AML class patients had a thrombotic manifestation at presentation [6]. Muñiz AE reported myocardial and cerebral ischemia as the presenting feature in an AML case [7]. Manea et al. presented a case of ischemic stroke as the presenting feature in the case of APML [8]. Our case was non-APML; we, however, could not perform a genetic analysis of the case to know the sub-type. Although rare, cerebral involvement in AML patients is associated with a five times higher mortality risk [9].

Hyperleukocytosis in the acute phase of the disease produces hyperviscosity causing blood stasis. Other factors related to thrombosis in acute leukemia are increased expression of tissue factors, endothelial activation, chemotherapy, sub-type of leukemia, and microparticles [10]. Microvasculature is most susceptible to these insults due to its small luminal diameter. It is a medical emergency that affects vital organs like the lungs, heart, and brain, needing site-specific and general measures like hydration and chemotherapy or leukapheresis [11].

A recent case report by Aoki et al. showed the feasibility of mechanical thrombo-embolectomy of Basilar artery occlusion [12]. Ischemic infarct presented within 4.5 hours of symptoms' onset showed a favorable outcome after thrombolysis [13]. In our case, the right middle cerebral arterial blockage had resulted in a massive cerebral infarct. The patient had a neurological deterioration >24h before presentation to a health care setup which excluded the possibility of thrombolysis. Moreover, thrombocytopenia also hindered the chance of thrombolysis [14]. Although recurrent transfusion of platelets was done, persistent thrombocytopenia was noted in our patient, possibly because of chemotherapy [15]. No standard management for acute ischemic stroke in AML patients with significant thrombocytopenia has been established. We started UFH considering the presentation time and risk of further thromboembolism, although its effect on the odds of being dead or dependent at final follow-up is doubtful [16]. A meta-analysis indicates functional outcome benefits from low molecular weight heparin (LMWH) when started after 24 hours of symptom onset. In contrast, increased intracranial hemorrhage was observed if LMWH started within 24 hours and was not recommended for early anticoagulation [17,18]. As our patient was intubated, passed 24h, and was immobilized, we initiated UFH to prevent venous thromboembolism. Related scientific organizations have also recommended using low-dose subcutaneous heparin or LMWH for immobilized patients (class I, level A) [18]. In the present case, the benefit of heparin use outweighed the risk of heparin-induced thrombocytopenia. Easy reversibility before taking up for surgery (decompressive craniotomy) was another advantage of UFH, which made our choice more prudent.

A recent study retrospectively evaluated 300 newly diagnosed cases of AML and found that platelet count >50,000/dL, presence of comorbidities, and previous thrombosis events were risk factors for having venous or arterial thrombosis in such patients [19]. Interestingly, our patient had none of these risk factors and presented with stroke as the presenting manifestation. 

## Conclusions

Thromboembolic events may be possible even before the diagnosis of AML, possibly due to hyperleukocytosis. Patients presenting beyond 24 hours of stroke onset may be treated with UFH or LMWH to prevent venous thromboembolism. Anticoagulant therapy may go hand-in-hand with chemotherapy for AML. However, the dilemma of decompressive craniotomy in the face of persistent thrombocytopenia and the need for an early restart of anti-coagulation for a massive cerebrovascular accident leading to midline shift and increased intracranial pressure continues.
